# Salubrious Effects of Green Tea Catechins on Fatty Liver Disease: A Systematic Review

**DOI:** 10.3390/medicines9030020

**Published:** 2022-03-01

**Authors:** Omar Abunofal, Chandra Mohan

**Affiliations:** Department of Biomedical Engineering, University of Houston, 3517 Cullen Blvd, Room 2004, Houston, TX 77204, USA; oabunofal@uh.edu

**Keywords:** non-alcoholic fatty liver disease, epigallocatechin-3-gallate, green tea extracts

## Abstract

Epigallocatechin-3-gallate (EGCG) is a polyphenol green tea catechin with potential health benefits and therapeutic effects in non-alcoholic fatty liver disease (NAFLD), a common liver disorder that adversely affects liver function and lipid metabolism. This systematic review surveyed the effects of EGCG or green tea extract (GTE) on NAFLD reported in studies involving rodent models or humans with a focus on clinicopathologic outcomes, lipid and carbohydrate metabolism, and inflammatory, oxidative stress, and liver injury markers. Articles involving clinical efficacy of EGCG/GTE on human subjects and rodent models were gathered by searching the PUBMED database and by referencing additional articles identified from other literature reviews. EGCG or GTE supplementation reduced body weight, adipose tissue deposits, and food intake. Mechanistically, the majority of these studies confirmed that EGCG or GTE supplementation plays a significant role in regulating lipid and glucose metabolism and expression of genes involved in lipid synthesis. Importantly, EGCG and GTE supplementation were shown to have beneficial effects on oxidative stress-related pathways that activate pro-inflammatory responses, leading to liver damage. In conclusion, green tea catechins are a potentially useful treatment option for NAFLD. More research is required to determine the ideal dosage, treatment duration, and most effective delivery method of EGCG or GTE, and to provide more definitive conclusions by performing large, randomized clinical trials.

## 1. Introduction

Non-alcoholic fatty liver disease (NAFLD) is a chronic hepatic disorder characterized by excessive lipid accumulation in the liver, which is not secondary to alcohol consumption. NAFLD can progress to non-alcoholic steatohepatitis (NASH), fibrosis, and eventual liver cirrhosis, hepatocellular carcinoma, and liver failure [1]. Patients in certain risk categories, including obesity, type II diabetes, hyperlipidemia, insulin resistance, and those who consume high-fat diets (HFDs) are particularly prone to NAFLD. Approximately 20–33% of adults in the United States have NAFLD, resulting in an estimated annual economic burden of $103 billion in direct costs [2,3].

Although there are no Food and Drug Administration-approved medications for NAFLD or NASH, some treatment strategies may reduce the manifestations of NAFLD. Diet and lifestyle modifications aid in limiting caloric intake, increasing physical activity, and improving liver histology. Various pharmacologic therapies regulate enzymatic activities in the liver, limit lipid formation, and prevent excessive inflammation and oxidative stress. However, most medications have had limited success or have substantial limitations, such as being unsustainable in long-term administration. Clinical trials of some medications failed to demonstrate high efficacy, whereas other studies evaluated only a small number of participants [4].

Green tea catechins are supplements that were widely studied over the past two decades for NAFLD. Green tea extracts (GTEs) are rich in flavonoids and possess prominent anti-inflammatory, antioxidative, and antilipidemic properties [5]. Epigallocatechin-3-gallate (EGCG) is the most commonly studied flavonoid because of its high abundance in green tea. Potential benefits of EGCG have been demonstrated in various in vitro and in vivo studies of animal models, and in various clinical trials of patients with NAFLD. In addition to its substantial benefits in NAFLD, EGCG also has positive effects in cancer, cardiovascular diseases, type II diabetes, and metabolic health, among others [6].

In earlier reviews of GTEs’ effects on NAFLD, Hodges et al. focused on articulating the anti-inflammatory mechanism of action of GTE through regulating the activation of nuclear factor-κB (NFκB) [7], focusing on human studies. Mahmoodi et al. examined the effects of GTE on liver enzymes from previous randomized clinical trials in NAFLD [8]. This manuscript extends beyond earlier reviews by providing a systematic review of the effects of EGCG and GTE observed in NAFLD in rodent and human studies, and by tabulating the effects of EGCG on clinicopathologic phenotypes, lipid and carbohydrate metabolism, inflammatory and oxidative stress markers, and liver enzymes, in a user-friendly format.

## 2. Methods

### 2.1. Literature Search and PRISMA Diagrams

**Figure 1:**
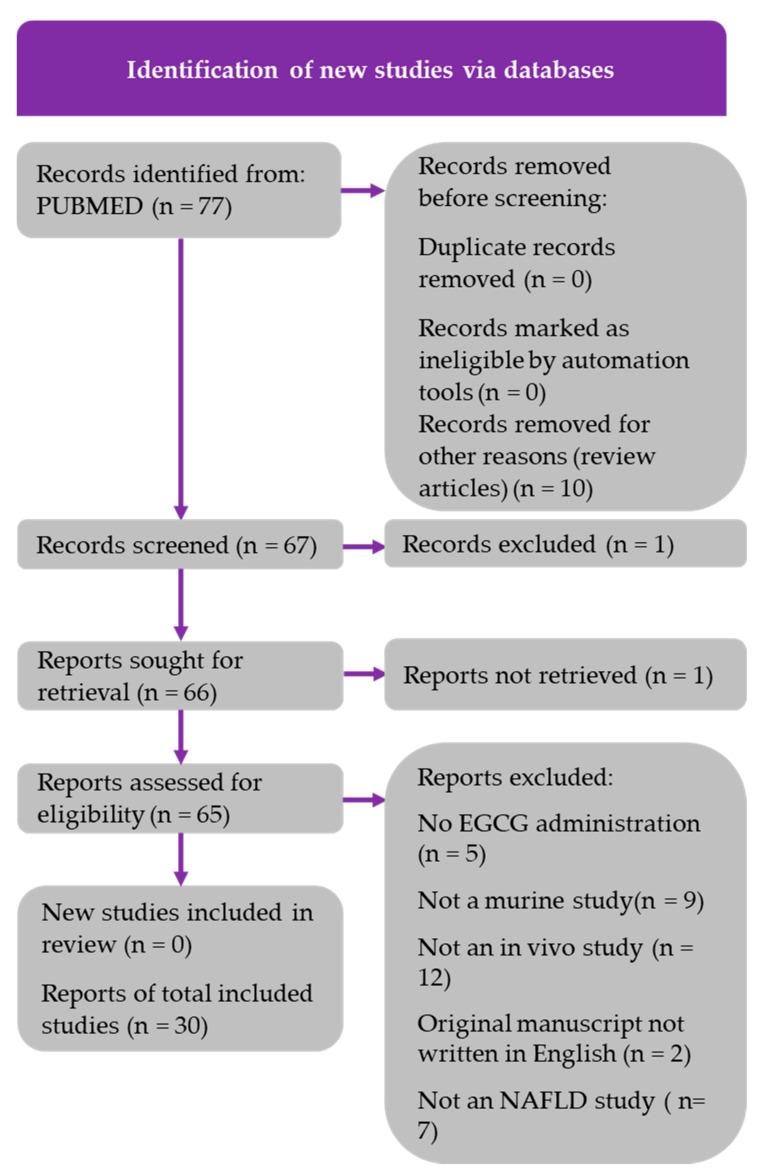
PRISMA flow chart for murine studies, indicating how the 30 studies were chosen.

**Figure 2:**
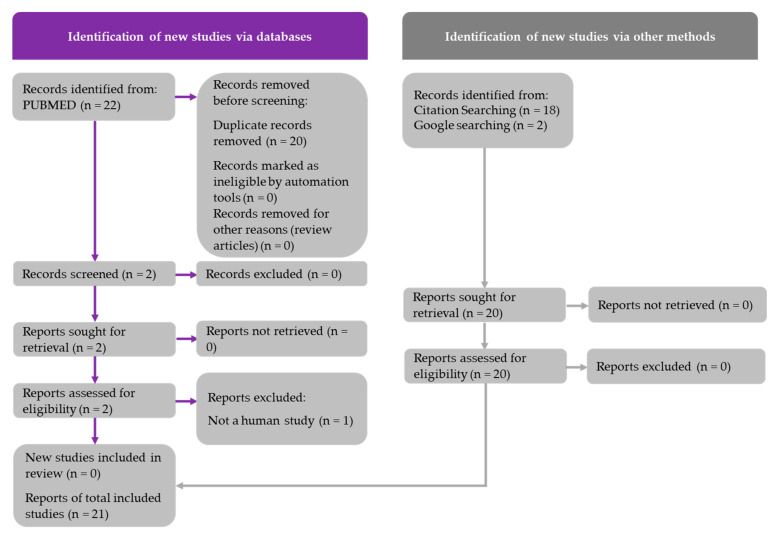
PRISMA flow chart for human studies indicating how the 21 studies were chosen.

Literature searches were conducted using the PubMed database to gather research articles involving EGCG or GTE use in humans and rodent models. The literature search was conducted on 16 December 2021, using the following search string for murine studies: (EGCG) AND (fatty liver OR NAFLD). The following search string was used for the human studies: (EGCG) AND (fatty liver OR NAFLD) AND (human studies). A total of 77 rodent studies and 22 human studies were retrieved from these searches. Additional human studies were identified by performing a Google search and by manually searching the reference lists of review articles, as indicated in Figure 1 and Figure 2. The article inclusion and data reporting were independently performed by one author, with no automation tools used during the process. PRISMA diagrams for both murine and human studies were constructed in accordance with the updated PRISMA 2020 guidelines. The inclusion criteria for the animal and human studies included the following: original articles must be written in English; a quantifiable dose of EGCG or GTE must be reported; studies must have sufficient study duration; in vivo studies are required; NAFLD must be induced in animal studies; and changes in clinical phenotypes must be reported. The PRISMA diagrams shown in Figure 1 and Figure 2 detail the search processes and final number of studies included in this systematic review (30 murine studies and 21 human studies). 

### 2.2. Risk of Bias Assessment

Risk of bias (ROB) assessment was performed in accordance with ‘Cochrane Risk of Bias Tool for Randomized Trials’ method. This method involves examining the studies on the basis of the trial design, conduct, and reporting through five different domains with specific questions in order to evaluate if a given study is at risk of bias or not. It was found that 16 of the 21 human studies examined for ROB assessment exhibited a low risk of bias, as these studies implemented effective study designs that best aligned with the context of the trial; the other 5 studies that exhibited moderate risk of bias were nevertheless included due to their eligibility in accordance with our inclusion criteria; moreover, the reported results were consistent with other studies that exhibited low risk of bias. All of the 21 human studies showed transparency with data reporting by clearly reporting the data of all parameters and reporting the difference from baseline.

## 3. Results

### 3.1. Findings from Rodent Studies

A wide range of analyses were performed in studies examining the effects of EGCG in rodent models. These studies explored clinicopathologic effects, lipid metabolism, carbohydrate metabolism, inflammatory markers, oxidative stress markers, and liver enzymes associated with EGCG treatment. In most studies, EGCG was delivered in the rodent chow. Table 1 summarizes the characteristics and findings of the 30 rodent studies included in this review.

#### 3.1.1. Clinicopathologic Effects

Body weight, liver weight, food intake, water intake, energy intake, and steatosis are common clinicopathologic metrics and metabolic risk factors. The majority of the studies reported a significant decrease in body weight after EGCG treatment [10,11,12,13,16,17,21,23,24,25,26,28,30,32,34,36,37,38] (Table 1). A total of nine studies reported a reduction in liver weight [11,12,17,23,24,26,29,34,38]. In 10 of 11 studies, EGCG supplementation was associated with a significant decrease in the mass of various types of adipose tissue [12,13,15,16,23,25,26,28,31,37]. Lee et al. reported that EGCG led to dose-dependent suppression of genes associated with adipogenesis, such as peroxisome proliferator receptor-γ (PPAR-γ) and CCAAT enhancer-binding protein-α (C/EBP-α) [13].

All studies examining the effects of EGCG on steatosis found that EGCG significantly reduced steatosis, ballooning, and inflammation scores (Table 1) [10,11,14,16,19,20,21,22,23,34,38]. Kuzu et al. reported decreases in steatosis and necrosis, associated with reduced α-smooth muscle actin (α-SMA) and cytochrome P450 2E1 (CYP2E1) levels [11]. Sumi et al. found that improvement of steatosis with EGCG was associated with inhibition of glutathione S-transferase-A placental form (GST-P)-positive foci, preneoplastic lesions associated with NAFLD [19]. Moreover, Gan et al. reported reduced steatosis accompanied by prominent hepatic cell regeneration, following EGCG administration [23].

#### 3.1.2. Lipid Metabolism

Many studies reported significant decreases in total cholesterol (TC), triglycerides (TG), and low-density lipoprotein (LDL) (Table 1). Significant decreases in TC were observed in 13 of 18 studies [9,13,14,23,26,28,29,30,31,34,36,37,38]; significant decreases in TG were reported in 18 of 22 studies [9,11,12,14,16,17,19,23,26,28,29,30,31,32,33,34,36,38]; and significant decreases in LDL were reported in 10 of 14 studies [9,13,23,26,27,28,29,31,36,37]. Thus, there is ample evidence indicating that EGCG exerts anti-hyperlipidemic effects.

**Figure 3:**
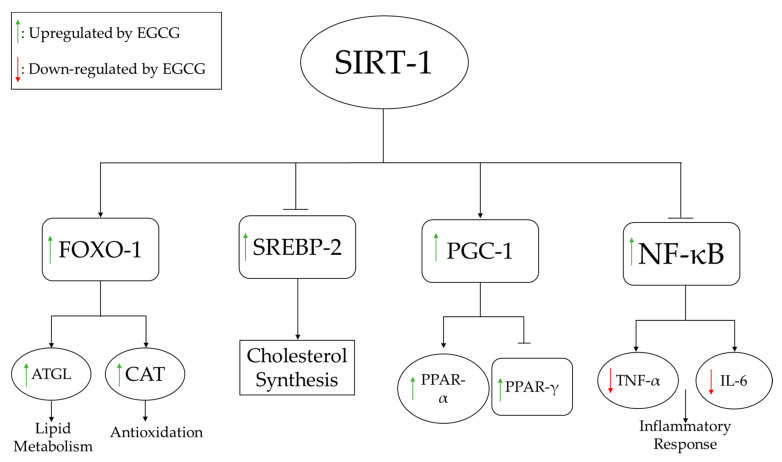
EGCG-induced SIRT-1 modulation of lipid metabolism, antioxidant pathways, inhibition of fatty acid synthesis, and inflammatory response pathways. ATGL increases lipolysis of fats, while increased CAT boosts the antioxidant status. NF-kB regulates several pro-inflammatory pathways, including the production of inflammatory cytokines such as IL-6 and TNFα. **Abbreviations: ATGL:** adipose triglyceride lipase; **CAT:** catalase; **FOXO-1:** fork-head box O1; **IL:** interleukin; **NF-κB:** nuclear factor-κB; **PGC:** peroxisome proliferator-activated receptor-gamma coactivator; **PPAR:** peroxisome proliferator receptor; **SREBP:** sterol regulatory element binding protein; **TNF:** tumor necrosis factor.

To understand the mechanisms underlying these lipid profile alterations, several pathways directly associated with lipid synthesis were evaluated at the molecular level. For example, Lee et al. examined the dose-dependent effects of EGCG on adipocyte differentiation genes and found that mRNA expression of PPARγ, C/EBP-α, lipoprotein lipase (LPL), and fatty acid synthase (FAS) decreased markedly following EGCG treatment and was highly correlated with a reduction of adipose tissue deposits [13]. Li et al. examined the effects of EGCG on several pathways, including the silent information regulator-1/forkhead box protein O1 (SIRT-1/FOXO1) pathway in conjunction with the regulatory gene, sterol regulatory element binding protein-2 (SREBP-2), which is responsible for regulating cholesterol synthesis [29]. As shown in Figure 3, EGCG supplementation activates SIRT-1, which increases the expression of FOXO1 and decreases the expression of SREBP-2. Increased FOXO1 expression induces lipid metabolism and increases antioxidant (catalase) activity, whereas decreased SREBP-2 expression reduces fatty acid synthesis.

#### 3.1.3. Carbohydrate Metabolism

It is important to consider the effects of EGCG on carbohydrate metabolism, as carbohydrate accumulation has detrimental effects on obesity and liver disease. Interestingly, all studies addressing the impact of EGCG on carbohydrate metabolism reported significant decreases in glucose and insulin levels, as well as insulin resistance (IR), with EGCG treatment [11,12,14,15,16,17,23,24,25,26,27,33,34,35,36,37] (Table 1). Gan et al. showed in their study that intraperitoneal administration of EGCG dose-dependently alleviates hyperinsulinemia, hyperglycemia, and IR [23]. The improvement of these parameters can be related to the weight loss reported by these authors.

#### 3.1.4. Inflammatory Markers

EGCG administration was shown to improve inflammatory profiles associated with liver damage. As shown in Table 1, 11 studies examined changes in inflammatory markers with EGCG administration [14,19,21,22,24,25,31,33,34,36,37], 7 of which reported that EGCG reduced inflammation by decreasing pro-inflammatory cytokines [21,22,24,33,34,36,37]. Yuan et al. found that EGCG improved inflammation by decreasing tumor necrosis factor-α (TNF-α) and interleukin-6 (IL-6) in obese rats, which extended the lifespan of the animals [36]. As shown in Figure 3, activation of SIRT-1 by EGCG leads to inhibition of NF-κB, thereby inhibiting production of TNF-α and IL-6.

#### 3.1.5. Oxidative Stress Markers

**Figure 4:**
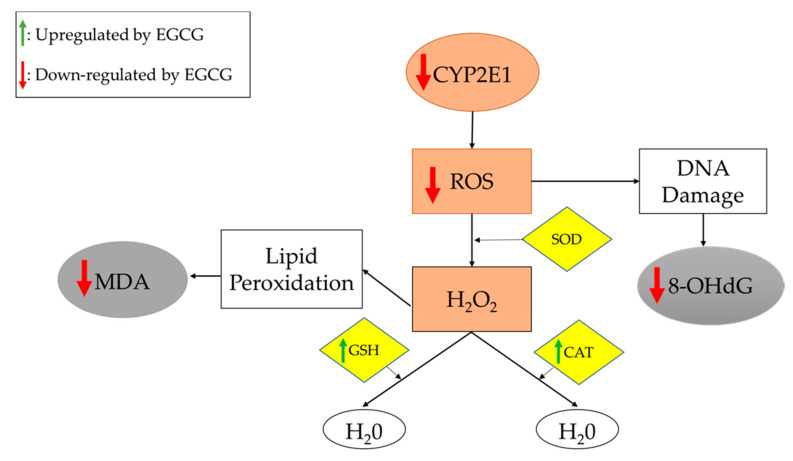
Oxidative stress and antioxidation pathways. Gray boxes represent pro-oxidation end-products. Brown boxes represent pro-oxidation molecules. Yellow boxes represent antioxidation molecules. Reducing the production of H_2_O_2_ or increasing its breakdown will reduce the oxidant stress. EGCG appears to act via both mechanisms. The end result is reduced oxidative damage, as is evidenced by the reduced levels of MDA and 8-OHdG. **Abbreviations: 8-OHdG:** 8-hydroxy-2’-deoxyguanosine; **CAT:** catalase; **CYP2E1:** cytochrome-2E1; **GSH:** glutathione; **MDA:** malondialdehyde; **ROS:** reactive oxidative species; **SOD:** superoxide dismutase.

Oxidative stress, a common feature in NAFLD, is facilitated by the accumulation of visceral fat, and contributes to lipid peroxidation that induces systemic oxidative damage [39]. Thus, assaying oxidative stress markers is useful for assessing NAFLD treatment effectiveness. Several important oxidative stress markers were investigated in the studies included in this review, as listed in Table 1. In 8 of 15 studies, EGCG administration was associated with significant decreases in pro-oxidants, such as CYP2E1 and reactive oxidative species (ROS), as well as oxidative end-products, such as 8-hydroxy-2’-deoxyguanosine (8-OHdG) and malondialdehyde (MDA) [11,14,19,20,24,29,34,36]. Furthermore, 6 of 10 studies reported significant increases in molecules involved in oxidation/reduction or detoxification reactions, including catalase (CAT), GST, and glutathione (GSH), upon EGCG administration [10,11,19,20,21,22]. Superoxide dismutase (SOD) is an enzyme that was evaluated in three studies in this review [21,24,36]. One study reported no changes in SOD [21], and the other two studies reported a significant increase in SOD [24,36], prompting the need for further studies. In this context, it should be pointed out that increased SOD may help remove superoxide radicals, while reduced SOD may also help by generating less H_2_O_2_ (see Figure 4).

Kuzu et al. examined the effects of EGCG on oxidative stress associated with the CYP2E1 enzyme. These authors concluded that EGCG potentially suppresses CYP2E1-associated oxidative stress, as it decreases lipid peroxidation and increases GSH levels [11]. As shown in Figure 4, EGCG treatment reduces the expression of CYP2E1, leading to decreased synthesis of ROS (which damage DNA) and increased expression of antioxidants, which alleviate oxidative stress.

#### 3.1.6. Biochemical Markers of Liver Damage

EGCG treatment also improves liver damage biomarkers [10,11,12,14,16,17,19,24,27,28,29,30,32,34,35,36,37,38]. As shown in Table 1, EGCG decreases levels of enzymes associated with liver damage, such as alanine aminotransferase (ALT), aspartate aminotransferase (AST), and alkaline phosphatase (ALP). Reductions in levels of these enzymes were associated with decreased steatosis.

### 3.2. Findings from Human Studies

As summarized in Table 2, 21 studies evaluating catechins, green tea, and EGCG or GTE in humans with NAFLD also evaluated clinicopathologic findings, lipid and carbohydrate metabolism, inflammation, oxidative stress, and liver damage. The majority of these studies conducted their clinical trials over a 12-week period, designed as randomized, double-blind, and placebo-controlled trials (Table 2).

#### 3.2.1. Clinicopathologic Effects

Administration of EGCG, GTE, and catechins was associated with a significant decrease in body weight in 9 of 15 studies [40,42,43,44,48,50,57,58,59] and a significant decrease in body mass index (BMI) in 7 of these 9 studies [42,43,44,50,57,58,59] (Table 2). Maki et al. examined the effects of green tea catechins on the body composition with obese adults and noted direct effects, similar to the findings in rodent studies [48].

Waist circumference (WC) was frequently evaluated as a surrogate for changes in body weight and fat loss. Five studies reported decreases in WC with catechins, EGCG, and GTE, concurrent with decreases in body weight [40,42,43,56,57]. Moreover, it is important to consider other possible mechanisms that may explain the decreases in body weight. For example, Chantre et al. found that GTE supplementation can inhibit gastric and pancreatic lipases, stimulate thermogenesis, increase energy expenditure (EE), and lower body weight; these changes can have substantial health benefits in obese patients [40]. In general, most studies did not find a change in energy intake or expenditure following treatment with EGCG/GTE (Table 2).

#### 3.2.2. Lipid Metabolism

Similar to the rodent studies, the human studies focused on LDL, TG, TC, and high-density lipoprotein (HDL) to evaluate the lipid metabolism effects of GTE (Table 2). Significant decreases in LDL were reported in 10 of 12 studies [42,43,46,50,52,54,55,56,57,59], and significant decreases in TC were reported in 6 of 12 studies [50,52,54,55,56,59]. For HDL, 4 of 12 studies reported a significant increase [46,50,55,59], but 7 of 12 studies reported no change [42,43,48,49,50,52,54,55,56]. Brown et al. reported no changes in LDL, HDL, TG, or TC in obese males who received 800 mg/day of oral EGCG [49]. When considering the results of other studies, these authors attributed the lack of improvement to the relatively low dosage of EGCG. They also noted that the peak EGCG plasma concentration was approximately 1 μM, suggesting low oral bioavailability. In general, adequate EGCG/GTE dosages appear to exert salubrious effects on lipid profiles, in resonance with findings from murine studies.

#### 3.2.3. Carbohydrate Metabolism

Human studies focused on investigating changes in glucose, insulin, and IR to evaluate the effectiveness of GTE in obese patients (Table 2). There were no significant changes in glucose levels in 8 of 12 studies [41,43,45,48,51,52,53,54], insulin levels in 7 of 11 studies [41,45,46,48,49,53,54], and IR in 3 of 6 studies [46,49,52]. Thus, these parameters were not significantly altered by tea catechins in most human studies, although they were significantly reduced in the rodent studies. Of note, the study duration may be important when considering alterations in metabolic syndrome markers. In reviewing the studies in Table 2, it appears that short-term studies reported no change in glucose and insulin, where treatment was administered for less than 6 weeks [53,54]. On the other hand, longer study durations were associated with changes in glucose, insulin, and IR [47,50,55,57]. Taken together, treatment with GTE should preferably be continued for at least 12 weeks, to observe effects on carbohydrate metabolites.

#### 3.2.4. Inflammatory Markers

As can be seen from Table 2, only five studies assayed inflammatory markers, with no conclusive trends [47,48,51,55,59]. Others reported that catechins have anti-inflammatory properties that suppress leukocyte adhesion to endothelium and inhibit transcription factors for cytokines and adhesion molecules, in other disease contexts. In contrast to rodent studies, few studies have examined the effects of EGCG on inflammatory markers in NAFLD, highlighting the need for more research examining inflammatory profiles in patients with NAFLD.

#### 3.2.5. Oxidative Stress Markers

An insufficient number of studies evaluated the effects of EGCG or GTE on oxidative stress markers. MDA and total antioxidant status (TAS) were the only oxidative stress markers assayed. MDA is the most frequently used biomarker of oxidative stress in various diseases [61]. TAS has an inverse relationship with other oxidative stress markers, such as MDA, as it represents antioxidative capacity [62]. Two of three studies reported significant decreases in MDA [42,52], and both studies investigating the effects of GTE on TAS reported significant increases [55,56]. Basu et al. reported a significant decrease in MDA, confirming the antioxidant properties of GTE, and Bogandaski et al. reported a significant increase in TAS after 3-month supplementation with GTE, indicating that GTE improved oxidative stress [52,55].

The antioxidative properties of green tea catechins are best appreciated by understanding the structural properties of EGCG. These properties have been attributed to the presence of dihydroxyl or trihydroxyl groups on the B-ring and meta-5,7-dihydroxyl groups on the A-ring. The polyphenolic structure of green tea catechins allows delocalization of electrons, which promotes the elimination of reactive oxygen and nitric radicals [56].

Although limited studies have examined the effects of EGCG or GTE in humans, the available data suggest that EGCG or GTE supplementation is a promising strategy for alleviating oxidative stress. The results of human studies are consistent with those of rodent studies, which clearly demonstrates the antioxidant effects of EGCG. Nevertheless, more research is necessary to confirm the efficacy of these supplements in reducing oxidative stress in humans.

#### 3.2.6. Liver Enzymes

Similar to rodent studies, serum AST and ALT were common metrics used for assessing liver damage in human studies, and both markers were decreased with EGCG and GTE treatment (Table 2). Following EGCG or GTE administration, significant decreases in AST were reported in four of six studies [47,57,59], and significant decreases in ALT were reported in three of five studies [47,58,59]. Pezeshki et al. reported significant decreases in AST and ALT following 12-week treatment with 500 mg/day of GTE [58]. This result was confirmed by Hussain et al., who found similar results using the same GTE dosage and treatment duration [59].

## 4. Discussion

EGCG is the most abundant and most extensively studied green tea catechin. A number of studies have demonstrated that EGCG and GTE are efficacious and have potential therapeutic benefits for NAFLD and other diseases. Anti-inflammatory, anti-oxidative, and anti-hyperlipidemic salubrious properties have been observed across both rodent and human studies using EGCG/GTE. Treatment for at least 12 weeks and using a daily EGCG or GTE dosage of 300–600 mg appears to be most beneficial in humans to observe substantial improvements in lipid profiles, oxidative status, and liver injury markers (Table 2).

In both rodent and human studies, green tea catechins reduced body weight, food intake, oxidative stress, and liver injury markers. EGCG aided in restoring lipid metabolism, which can be associated with reductions in body weight and food intake. Rodent studies showed that EGCG treatment can reduce levels of pro-oxidative molecules, such as CYP2E1 and α-SMA, and thereby decrease inflammation. Inflammation and oxidative stress are hallmarks of NAFLD and many other diseases. Oxidative stress can generate free radicals that cause direct organ damage and activate inflammatory pathways [63]. EGCG was shown to decrease pro-inflammatory cytokines and pro-oxidative stress molecules in both human and rodent studies, and also increased the production of SIRT-1. As summarized in Figure 3, SIRT-1 activates several pathways that inhibit the production of pro-inflammatory cytokines and fatty acids, as well as increase lipid metabolism and the production of antioxidants [64]. Pro-oxidation molecules, such as CYP2E1 and ROS, activate pathways for lipid peroxidation and DNA damage that can lead to oxidative stress, as shown in Figure 4. EGCG supplementation can promote the production of antioxidants that reign in oxidative stress.

Taken together, the findings of this review indicate that EGCG and GTE are efficacious natural substances that aid in reducing multiple manifestations of NAFLD by regulating lipid and carbohydrate metabolism, attenuating inflammation and oxidative stress, and reducing liver cell damage, as indicated by decreased liver injury markers. Treatment duration and EGCG dose appear to have substantial effects on outcomes, with longer durations and appropriate dosage being necessary for optimal treatment outcomes.

In conclusion, this systematic review re-affirms EGCG and GTE as potentially promising therapeutic options for NAFLD. Current treatment options for this disorder include physical exercise, dietary modifications, and experimental therapies. In some instances, the latter can be associated with adverse events, as documented for pioglitazone, which has been linked to increased risks of prostate or pancreatic cancer, fluid retention, bone fractures, and cardiovascular events [65].

Several aspects of this study are limiting. First, no attempt was made to standardize or normalize data from all included studies so that similar reporting instruments and metrics are used across all studies. Second, no attempt was made to do a meta-analysis of combined data from the individual studies. Finally, not all papers assessed all clinical and laboratory outcomes comprehensively; hence, some of the conclusions drawn from this review may need to be revised once more data becomes available.

Currently, there are no FDA approved medications for NAFLD, though several candidates are in various stages of clinical trials, with a wide range of efficacies and adverse events [66]. Physical exercise plays a role in the alleviation of NAFLD and NASH, as it has been shown to reverse the progression of NAFLD [67]. Against this backdrop, EGCG and GTE appear to be efficacious alternatives, with minimal side effects. Given the promising findings, further research is required to dissect out the mechanistic modes of action of EGCG and GTE using modern day OMICs based technologies including transcriptomics, proteomics, and metabolomics.

## Figures and Tables

**Table 1:** Clinical Efficacy of EGCG Supplementation in Rodent Models.

Study [Ref]	Model	EGCG Intake	Duration	Clinical/Pathological Outcome	Lipid Metabolism	Carbohydrate Metabolism	Inflammatory Markers	Oxidative Stress Markers	Liver Injury Enzymes
Raederstorff 2003 [9]	HFD (R)	0.25–1% (CD)	4 weeks	↑ Fecal fat/cholesterol/lipid excretion; ↔ Body weight, liver weight, food intake	↓ TC, LDL, HDL, TG				
Fiorini 2005 [10]	I/R (M)	85 mg/kg (DW/IP)	5 days	↓ Body weight, steatosis; ↔ Food intake	↓ FAS			↑ GSH; ↔ UCP	↓ ALT
Kuzu 2007 [11]	HFD (R)	1 g/L (DW)	6 weeks	↓ Body weight, liver weight, steatosis, inflammation; ↔ Degeneration, necrosis	↓ TG; ↔ TC	↓ Insulin, IR		↓ MDA, CYP2E1; ↑ GSH	↓ ALT; ↔ ALP, AST
Bose 2008 [12]	HFD (M)	3.2 g/kg (CD)	16 weeks	↓ Body weight, liver weight, MAT, VAT, EAT, RAT	↓ TG; ↑ Fecal lipids	↓ Glucose, insulin, IR			↓ ALT
Lee 2008 [13]	HFD (M)	0.2–0.5% (CD)	8 weeks	↓ Body weight, EAT, VAT, RAT; ↔ Liver weight, energy intake	↓ TC, LDL, PPAR-γ, FAS, LPL; ↑ CPT-I, HSL, ATGL			↑ UCP-II	↔ ALT, AST
Ueno 2009 [14]	NASH (M)	0.05–0.1% (DW)	42 weeks	↓ Steatosis, intralobular fibrosis, ballooning; ↔ Body weight	↓ TC, TG; ↔ FFA	↓ Glucose	↓ pAkt, pIKKß, pNF-κB	↓ 8-OhdG	↓ ALT; ↔ AST
Chen 2009 [15]	HFD (R)	1 mg/kg (DW)	23 weeks	↓ WAT;↑ Body weight; ↔ Food intake	↑ PPAR-γ; ↔ TC, LDL, HDL, TG, SREBP-1C, PPAR-α, CPT-II, FAS, ACC	↓ Glucose		↑ UCP-II; ↔ ACO, MCD	
Chen 2011 [16]	HFD (M)	0.32% (CD)	17 weeks	↓ Body weight, BAT, steatosis; ↔ Food intake	↓ TG; ↑ Fecal lipids	↓ Glucose, insulin, IR			↓ ALT, HSL
Sae-tan 2011 [17]	HFD (M)	0.32% (CD)	15 weeks	↓ Body weight, liver weight; ↔ Food intake	↓ TG	↓ Glucose, insulin			↓ ALT
Sugiura 2012 [18]	HFD (M)	0.1% (DW)	4 weeks	↔ Body weight, liver weight, food intake, IPAT	↔ TC, TG, FAS, CPT-II			↔ ACO	
Sumi 2013 [19]	HFD (R)	0.01–0.1% (DW)	7 weeks	↓ Liver fibrosis, steatosis; ↔ Body weight, liver weight	↓ TG		↑ TNF-α, IL-6	↓ GPx-1, GST-P+, 8-OHdG, d-ROM; ↑ CAT	↓ ALT
Kochi 2013 [20]	HFD (R)	0.1% (DW)	9 weeks	↓ Steatosis; ↑ Body weight				↓ MDA, 8-OHdG, GST-P+, d-ROM, CYP2E1; ↑ GPx, CAT	
Xiao 2013 [21]	HFD (R)	50 mg/kg (IP)	8 weeks	↓ Body weight, food intake, steatosis, fibrosis			↓ TNF-α, COX-2	↑ GPx, CAT; ↔ SOD	
Krishnan 2014 [22]	HFD (R)	100 mg/kg (OG)	30 days	↓ Steatosis, inflammation			↓ NF-κB, TNF-α		
Gan 2015 [23]	HFD (M)	10–40 mg/kg (IP)	24 weeks	↓ Energy intake, body weight, liver weight, steatosis, VAT; ↑ Hepatic cells	↓ TC, TG, LDL; ↑ HDL	↓ Glucose, insulin, IR, glucose intolerance			
Ding 2015 [24]	MCDD (M)	25–100 mg/kg (IP)	4 weeks	↓ Body weight, liver weight, food intake			↓ IL-1β, IL-6, TNF-α, MCP-1	↓ MDA; ↑ SOD	↓ AST, ALT
Santamarina 2015 [25]	HFD (M)	50 mg/kg (DW)	16 weeks	↓ Body weight, WAT, ectopic fat, MAT; ↔ Liver weight, EAT, RAT		↓ Glucose, insulin, IR	↔ TNF-α, IL-6, IL-10, IL-6R, IL-10Rα		
Mi 2017 [26]	HFD (M)	2 g/L (DW)	16 weeks	↓ Body weight, liver weight, BAT	↓ TG, TC, LDL; ↑ HDL, PPAR-γ, ACC, SIRT-I, FAS, SREBP-1C, CPT-II, CPT-Iα	↓ Glucose, insulin, IR; ↑ Glucose tolerance, insulin sensitivity			
Huang 2018 [27]	HFD (M)	3.2 g/kg (CD)	33 weeks	↔ Body weight, liver weight, food intake	↓ LDL; ↑ HDL, HMGCR, PPARα; ↔ TG, FAS	↓ Glucose		↑ CYP7A1, CYP27A1	↓ ALT
Yang 2018 [28]	HFD (R)	160 mg/kg (OG)	11 weeks	↓ Body weight, WAT, energy intake	↓ TC, LDL, HDL, TG, NEFA				↓ ALT, AST
Li 2018 [29]	HFD (R)	25–100 mg/kg (CD)	4 weeks	↓ Liver weight	↓ TC, LDL, TG, FFA, SREBP-II; ↑ HDL, SIRT-I, FOXO-I; ↔ HMGCR			↓ MDA	↓ ALT, AST
Sheng 2018 [30]	HFD (M)	100 μg/g (CD)	8 weeks	↓ Body weight	↓ TC, TG; ↔ LPL				↓ ALP, ALT
Li 2018 [31]	HFD (m)	50–100 mg/kg (IG)	20 weeks	↓ EAT; ↔ Body weight	↓ LDL, TC, TG, CPT1α; ↑ HDL, ACC, FAS, ATGL		↓ PPARα, ACO2; ↑ PPARγ, SREBP1	↓ UCP2	↑ HSL
Ushiroda 2019 [32]	HFD (M)	0.32% (CD)	24 weeks	↓ Body weight; ↔ Food intake	↓ TG; ↔ LDL, HDL, TC, NEFA				↓ ALT, AST
Hou 2020 [33]	HFD (R)	0.32% (CD)	16 weeks	↔ Body weight	↓ FFA, TG, IR	↓ IR	↓ TNF-α, p-NF-κb, TRAF6, IKKβ, p-IKKβ, TLR4		
Dey 2020 [34]	HFD (M)	0.3% (CD)	8 weeks	↓ Body weight, liver weight, steatosis, ballooning; ↑ Energy intake	↓ TC, TG; ↔ NEFA	↓ Glucose, insulin, IR	↓ TLR4, NF-κb, MCP-1, TNF-α	↓ MDA	↓ ALT
Ning 2020 [35]	MCDD (M)	50 mg/kg (IP/OG)	2 weeks	↔ Body weight	↔ LDL, HDL, TC, TG	↓ Glucose			↓ ALT; ↔ AST
Yuan 2020 [36]	HFD (R)	50 mg/kg (DW)	92 weeks	↓ Body weight; ↔ Food intake	↓ TC, TG, LDL, FFA; ↔ HDL; ↑ CPT-II, FOXO1, SIRT1, FAS, ACC	↓ Glucose, insulin	↓ IL-6, TNF-α; ↑ NF-κB	↓ ROS; ↑ CAT, SOD; ↔ MDA	↓ ALT, AST
Huang 2020 [37]	HFD (M)	0.4% (CD)	14 weeks	↓ Body weight, EAT, PAT, MAT; ↔ Food intake	↓ TC, LDL	↓ Glucose	↓ TNF-α, IL-6, LPS, MMP-3, COX-2, TLR4		↓ ALT, AST
Du 2021 [38]	HFD (M)	25–50 mg/kg (CD)	16 weeks	↓ Body weight, liver weight, steatosis	↓ TG, HDL, TC; ↔ LDL				↓ AST, ALT

↑↓ indicates an increase or decrease in the value of the respective variable. **↔** indicates that no change occurred in that respective variable. Green font represents parameters that were increased; red font represents parameters that were decreased; blue font represents parameters that did not change, following EGCG treatment. **Abbreviations: 8-OHdG:** 8-hydroxy-2’-deoxyguanosine; **ACC:** acetyl CoA carboxylase; **ACO:** acyl-CoA oxidase; **AKT:** protein kinase B; **ALP:** alkaline phosphatase; **ALT:** alanine aminotransferase; **AST:** aspartate aminotransferase; **ATGL:** adipose triglyceride lipase; **BAT:** brown adipose tissue; **CAT:** catalase; **CD:** chow diet; **COX:** cyclooxygenase; **CPT:** carnitine palmitoyl transferase; **CYP:** cytochrome; **d-ROM:** derivatives of reactive oxygen metabolites; **DW:** drinking water; **EAT:** epididymal adipose tissue; **EGCG:** epigallocatechin-3-gallate; **ET:** endotoxin; **FAS:** fatty acid synthase; **FFA:** free fatty acid; **FOXO1:** fork-head box O1; **GLUT4:** insulin-regulated glucose transporter; **GPx:** glutathione peroxidase; **GSH:** glutathione; **GST-P+:** glutathione S-transferase–positive; **HDL:** high-density lipoprotein; **HFD:** high-fat diet; **HMGCR:** 3-hydroxy-3-methylglutaryl coenzyme A reductase; **HSL:** hormone-sensitive lipase; **IG:** intragastric; **IKK:** inhibitor of nuclear factor-κB kinase; **IL:** interleukin; **IP:** intraperitoneal; **IPAT:** intraperitoneal adipose tissue; **IR:** insulin resistance; **I/R:** ischemia/reperfusion; **LDL:** low-density lipoprotein; **LPL:** lipoprotein lipase; **LPS:** lipopolysaccharide; **M:** mouse; **MAT:** mesenteric adipose tissue; **MCD:** malonyl CoA decarboxylase; **MCDD:** methionine-and choline-deficient diet; **MCP:** monocyte chemoattractant protein; **MDA:** malondialdehyde; **MMP:** matrix metalloproteinases; **NASH:** non-alcoholic steatohepatitis; **NEFA:** non-esterified fatty acid; **NF:** nuclear factor; **OG:** oral gavage; **PAT:** peritoneal adipose tissue; **PPAR:** peroxisome proliferator receptor; **R:** rat; **RAT:** retroperitoneal adipose tissue; **SIRT:** sirtuin; **SOD:** superoxide dismutase; **SREBP:** sterol regulatory element binding protein; **TC:** total cholesterol; **TG:** triglycerides; **TLR:** toll-like receptor; **TNF:** tumor necrosis factor; **TRAF:** tumor necrosis factor receptor-associated factor; **UCP:** uncoupling protein; **VAT:** visceral adipose tissue; **WAT:** white adipose tissue.

**Table 2:** Clinical Efficacy of Green Tea in Human Studies.

Study (Ref)	Study Design	Duration (Number of Participants)	Green Tea Component Daily Intake	Clinical/Pathological Outcome	Lipid Metabolism	Carbohydrate Metabolism	Inflammatory Markers	Oxidative Stress Markers	Liver Injury Enzymes
Chantre 2002 [40]	Open study	12 weeks (70)	375 mg catechin	↓ Body weight, WC	↔TC				
Kovacs 2003 [41]	(R/P/PC)	13 weeks (104)	323 mg EGCG	↔ Body weight, BMI, REE, RQ	↔ TG, NEFA	↔ Glucose, insulin			
Nagao 2004 [42]	(DB)	12 weeks (38)	690 mg catechin	↓ Body weight, BMI, WC, HC	↓ LDL; ↑ FFA; ↔ HDL, TG	↑ Glucose, insulin		↓ MDA	
Nagao 2006 [43]	(R/DB)	12 weeks (240)	583 mg catechin	↓ Body weight, BMI, WC, HC; ↔ Energy intake	↓ LDL; ↔ HDL, TC, TG, FFA	↔ Glucose			↔ ALP
Auvichayapat 2007 [44]	(R)	12 weeks (60)	750 mg green tea	↓ Body weight, BMI; ↑ REE; ↔ Food intake, physical activity, RQ					
Hill 2007 [45]	(R/PC)	12 weeks (38)	300 mg EGCG	↓ Total body fat, WC; ↔ Body weight, energy intake, EE, BMI, HC		↔ Glucose, insulin			
Hsu 2008 [46]	(R/DB/PC)	12 weeks (78)	1200 mg GTE	↓ WC, HC; ↔ Body weight, BMI	↓ LDL, TG; ↑ HDL; ↔ TC	↔ Insulin, IR			↔ AST
Matsuyama 2008 [47]	(R/DB)	36 weeks (40)	75–576 mg catechins	↔ Body weight, BMI, HC	↓ TG, FFA	↓ Glucose	↑ CRP		↓ AST, ALT
Maki 2008 [48]	(R/DB/C)	12 weeks (107)	625 mg EGCG	↓ Body weight; ↔ Physical activity, energy intake, WC	↓ TG, FFA; ↔ LDL, HDL	↔ Glucose, insulin	↔ CRP	↔ MDA	
Brown 2009 [49]	(R/DB/PC/P)	8 weeks (88)	800 mg EGCG	↔ BMI, WC	↔ TC, HDL, LDL, TG	↔ Insulin, IR			
Pierro 2009 [50]	(R)	90 days (100)	300 mg GTE	↓ Body weight, BMI	↓ TG, LDL, TC; ↑ HDL	↓ Glucose, insulin			
Basu 2010 [51]	(R/C)	8 weeks (35)	440 mg EGCG	↔ WC	↔ TG, HDL	↔ Glucose	↔ IL-6, IL-1β, sVCAM-1, CRP		↔ AST, ALT
Basu 2010 [52]	(R/C/SB)	8 weeks (35)	900 mg EGCG in capsule	↔ Body weight, BMI, WC	↓ TC, LDL; ↔ TG	↔ Glucose, IR		↓ MDA	
Thielecke 2010 [53]	(R/DB/PC/X)	3 days (12)	300–600 mg EGCG in capsule	↔ EE, RQ	↔ NEFA	↔ Glucose, insulin			
Brown 2011 [54]	(R/PC/X)	6 weeks (70)	800 mg catechins	↑ Energy intake; ↔ Body weight	↓ LDL; ↔ HDL, TG	↔ Glucose, insulin			
Bogdanski 2011 [55]	(DB/PC)	3 months (56)	379 mg GTE	↔ BMI, WC	↓ TC, LDL, TG; ↑ HDL	↓ Glucose, insulin, IR	↓ CRP, TNF-α	↑ TAS	
Suliburska 2012 [56]	(R/DB/PC/C)	3 months (46)	379 mg GTE	↓ BMI, WC	↓TC, LDL, TG; ↔ HDL	↔ Glucose		↑ TAS	
Mielgo-Ayuso 2013 [57]	(R/DB/PC)	12 weeks (88)	300 mg EGCG	↓ Body weight, BMI, WC	↓ TC, LDL, HDL; ↔ TG	↓ Insulin, IR			↓ AST; ↔ ALT
Pezeshki 2016 [58]	(R/DB/PC)	90 days (80)	500 mg GTE	↓ Body weight, BMI					↓ AST, ALT, ALP
Hussain 2017 [59]	(R/PC)	91 days (80)	500 mg GTE	↓ Body weight, BMI	↓ TC, LDL, TG; ↑ HDL	↓ IR	↓ CRP		↓ AST, ALT
Roberts 2021 [60]	(R/DB/PC)	8 weeks (27)	580 mg GTE	↔ Body weight, BMI, EE, WC	↔ TC, TG, LDL, HDL, FFA				↔ALT, AST, ALP

**↑↓** indicates an increase or decrease in the value of the respective variable. **↔** indicates that no change occurred in that respective variable. Green font represents the parameters that were increased; red font represents the parameters that were decreased; blue font represents parameters that did not change, following EGCG treatment. **Abbreviations: ALP:** alkaline phosphatase; **ALT:** alanine aminotransferase; **AST:** aspartate aminotransferase; **BMI:** body mass index; **C:** controlled; **CRP:** C-reactive protein; **DB:** double-blind; **EE:** energy expenditure; **EGCG:** epigallocatechin-3-gallate; **FFA:** free fatty acid; **GTE:** green tea extract; **HC:** hip circumference; **HDL:** high-density lipoprotein; **IL:** interleukin; **IR:** insulin resistance; **LDL:** low-density lipoprotein; **MDA:** malondialdehyde; **NEFA:** non-esterified fatty acid; **P:** parallel; **PC:** placebo controlled; **R:** randomized; **REE:** resting energy expenditure; **RQ:** respiratory quotient; **sVCAM:** circulating vascular adhesion molecule; **TAS:** total antioxidant status; **TC:** total cholesterol; **TG:** triglycerides; **TNF:** tumor necrosis factor; **WC:** waist circumference; **X:** cross-over trial.

## Data Availability

Not applicable.
